# The Frequency of Undiagnosed Celiac Disease in Youth with Type 1 Diabetes and Its Association with Diabetic Retinopathy: The SEARCH for Diabetes in Youth Study

**DOI:** 10.1155/2023/9038795

**Published:** 2023-05-29

**Authors:** Ryan P. Brady, Elizabeth T. Jensen, Joseph Rigdon, Nancy A. Crimmins, Daniel Mallon, Lawrence M. Dolan, Giuseppina Imperatore, Anna R. Kahkoska, Amy K. Mottl, Ann Honor, David J. Pettitt, Lina Merjaneh, Dana Dabelea, Amy S. Shah

**Affiliations:** ^1^Department of Pediatrics, Cincinnati Children's Hospital & University of Cincinnati College of Medicine, Cincinnati, OH 45229, USA; ^2^Department of Epidemiology and Prevention, Wake Forest School of Medicine, Winston-Salem, NC 27157, USA; ^3^Department of Biostatistics and Data Science, Wake Forest School of Medicine, Winston-Salem, NC 27157, USA; ^4^Division of Diabetes Translation, Centers for Disease Control and Prevention, Atlanta, GA 30341, USA; ^5^Department of Nutrition, University of North Carolina at Chapel Hill, Chapel Hill, NC 27599, USA; ^6^Department of Medicine, University of North Carolina at Chapel Hill, Chapel Hill, NC 27599, USA; ^7^Department of Pediatrics, Wake Forest School of Medicine, Winston-Salem, NC 27157, USA; ^8^Santa Barbara, CA 93103, USA; ^9^Department of Pediatrics, University of Washington, Seattle, WA 98195, USA; ^10^Lifeourse Epidemiology of Adiposity and Diabetes (LEAD) Center, Departments of Epidemiology and Pediatrics, University of Colorado Anschutz Medical Campus, Aurora, CO 80045, USA

## Abstract

**Aims:**

Celiac disease (CD) in adults with type 1 diabetes has been associated with increased cardiovascular risk and the earlier occurrence of diabetes-associated complications. In the Search for Diabetes in Youth study, we aimed to assess the frequency of CD and the potential for undiagnosed CD among youth with childhood onset type 1 diabetes. In addition, we assessed the burden of cardiovascular risk factors and diabetes-associated complications in youth with type 1 diabetes by CD status and IgA tissue transglutaminase autoantibody (tTGA) levels.

**Methods:**

2,444 youths with type 1 diabetes completed a CD questionnaire and underwent tTGA testing. Integrating the celiac disease questionnaire and tTGA results for this cross-sectional analysis, participants were categorized as follows: (1) reported CD; (2) seropositive for CD (no reported CD and seropositive tTGA); and (3) type 1 diabetes only (comparison group: no reported CD and seronegative tTGA). Subanalyses were performed on those with no reported CD and tTGA ≥10x ULN, designated potentially undiagnosed CD. Cardiovascular risk factors and diabetes-associated complications were evaluated by CD status and tTGA levels utilizing a Poisson model to estimate relative risk.

**Results:**

Reported CD in youths with type 1 diabetes was 7%. Seropositivity for tTGA with no reported CD was present in 4%, and 1.2% had potentially undiagnosed CD. Youths with potentially undiagnosed CD had a 2.69x higher risk of diabetic retinopathy than comparison group. In addition, CD with tTGA <0.05 (controlled CD) was associated with lower HbA1c.

**Conclusions:**

Undiagnosed CD is likely present in youths with type 1 diabetes and potentially undiagnosed CD is associated with a higher risk of diabetic retinopathy. These findings indicate the importance of routine screening for CD in type 1 diabetes in youths.

## 1. Introduction

Celiac disease (CD) is a chronic and inflammatory enteropathy triggered by the ingestion of gluten in genetically predisposed individuals [1]. CD and type 1 diabetes are both autoimmune diseases with shared genetic risk [2], and their association has been well described [3, 4]. While routine screening guidelines for CD in youth with type 1 diabetes have been published [5], given the often asymptomatic nature of CD in this population [6], it has the potential to remain undiagnosed.

CD in adults with type 1 diabetes is associated with higher cardiovascular risk [7–10], the earlier occurrence of diabetes-associated complications [11], and increased mortality [12]. As both disease processes often begin in childhood [13] and have been increasing in prevalence over time [14, 15], there is concern cardiovascular risk factors and the antecedents of diabetes-associated complications may occur in youth. Early diagnosis of CD and subsequent interventions therefore may reduce future cardiovascular burden.

The objectives of this study were to assess the frequency and characteristics of CD and the potential for undiagnosed CD in youths with type 1 diabetes who were enrolled in the Search for Diabetes in Youth study, a large observational cohort of youths with childhood onset diabetes in the United States. In addition, we assessed the burden of cardiovascular risk factors and diabetes-associated complications by CD status and IgA tissue transglutaminase autoantibodies (tTGA) levels. Developing a better understanding of the frequency and added disease burden of CD, particularly undiagnosed CD, will help guide existing screening practices in youth with type 1 diabetes. Our hypotheses were that CD may be undiagnosed in the youth-onset type 1 diabetes population and that CD is associated with cardiovascular risk factors and diabetes-associated complications at a young age.

## 2. Materials and Methods

### 2.1. Study Participants

Participants included in this study were enrolled in the Search for Diabetes in Youth study, a multicenter study covering 5 geographically distinct areas (California, Colorado, Ohio, South Carolina, and Washington) of the United States investigating the prevalence, incidence, and complications in participants with youth-onset diabetes (onset before 20 years of age). Comprehensive details of the SEARCH study have been published [16]. Individuals included in this analysis had a diagnosis of type 1 diabetes based on medical record review or referring physician [16] and/or had at least one diabetes autoantibody detectable in their serum at the time of their baseline SEARCH study visit.

Participants for the current study met the following additional inclusion criteria: (1) a baseline search visit in 2002–2006 or 2008 [16]; (2) a follow-up study visit in the years 2011 to 2019; and (3) duration of diabetes >5 years at the follow-up visit. At the follow-up visit, participants were assessed for the presence of CD, cardiovascular risk factors, and diabetes-associated complications and comorbidities [16]. The study was reviewed and approved by the local institutional review boards at each of the 5 SEARCH study sites and all participants and their parents/guardians provided informed assent and/or consent.

### 2.2. Clinical, Demographic, and Biochemical Assessment

Medical history and demographic information were obtained through questionnaires completed by study participants and/or their parents/guardians. Presence of autoimmune conditions (possible answers were “yes,” “no,” and “do not know”) including hyperthyroidism, hypothyroidism, Addison's disease, and vitiligo were ascertained by questionnaire. Additional autoimmune conditions (e.g., arthritis) were not ascertained at SEARCH study visits and therefore not available. Sex, race, and ethnicity were self-reported, and race and ethnicity were categorized as Hispanic, non-Hispanic Black (NHB), non-Hispanic White (NHW), or other. Resting systolic blood pressure (SBP) and diastolic blood pressure (DBP) were measured 3 times using an aneroid sphygmomanometer and an appropriately sized cuff by trained study staff. Participants were seated for 5 minutes prior to measurements and the average of the 3 measurements was used. Venipuncture was performed after at least an 8-hour overnight fast. Biochemical measurements of HbA1c and lipids were performed as described previously [16].

### 2.3. Celiac Disease Assessment

The presence of CD [5] in the SEARCH study was assessed using questionnaire response. The questionnaire asked whether a health care provider ever informed the participant that they had CD (possible answers were “yes,” “no,” and “do not know”). Those who answered, “do not know” (*n* = 27) were excluded from further analysis. tTGA were measured for this study utilizing stored serum samples from the SEARCH biospecimen repository on the remaining 2,444 participants with type 1 diabetes. The stored serum samples used to assess tTGA levels were collected at the time of the study follow-up visit and therefore coincide temporally with all data (questionnaire, diabetes complication, anthropometric, and biochemical data) reported here. Radiobinding assay using recombinant tissue transglutaminase protein was used to detect tTGA [17, 18]. tTGA assays were performed at the Barbara Davis Center Autoantibody Core Facility (Denver, CO, USA), which is a Clinical Laboratory Improvement Amendments certified laboratory and has been designated as a National Institutes of Health/National Institute of Diabetes and Digestive and Kidney Diseases North America Autoantibody Core Laboratory [19]. The assay result is expressed as an index with an assay range of −0.100 to 2.000. A tTGA level of ≥0.05 was used as the cut-off for a positive test (100th percentile of normal controls) [13, 17], and all levels >0.030 were repeated using the same assay to confirm a positive result.

### 2.4. Celiac Disease Status

First, we integrated the questionnaire response for reported CD and the laboratory measured tTGA level for all participants with type 1 diabetes to establish the following three mutually exclusive categories for analysis of CD status:

1. Individuals with reported CD: participants who answered “yes” to the CD question in the questionnaire, regardless of tTGA levels
2. Individuals seropositive for CD: participants who answered “no” to the CD question but had seropositive tTGA (levels ≥0.05)
3. Individuals with type 1 diabetes only (serving as comparison group): participants who answered “no” to CD question and had seronegative tTGA (levels <0.05)

Of the individuals categorized as seropositive for CD (abovementioned group 2), subanalysis was performed on those with tTGA ≥0.50 (≥10x ULN). These individuals, who had no reported CD, were designated for the purpose of this analysis as those with “potentially undiagnosed CD” based on two prior studies demonstrating that all individuals in their studies with tTGA levels ≥10 ULN had diagnosed CD after intestinal biopsy [20, 21]. In addition, using the same tTGA assay performed here, one study found a single tTGA level ≥10 ULN at the time of intestinal biopsy had a 96% positive predictive value (PPV) for positive intestinal biopsy specimen [22].

Second, we also sought to examine differences in cardiovascular risk factors among participants by tTGA levels. For this, four mutually exclusive categories were established:

1. Controlled CD: participants who answered “yes” to CD question and had seronegative tTGA levels (levels <0.05)
2. Intermediate tTGA elevation: all participants who had seropositive tTGA levels between ≥0.05 and <0.50, regardless of CD questionnaire response
3. High tTGA elevation: all participants with seropositive tTGA levels ≥0.50 (10x ULN), regardless of CD questionnaire response
4. Individuals with type 1 diabetes only (serving as comparison group): participants who answered “no” to CD question and had seronegative tTGA (levels <0.05)

### 2.5. Cardiovascular Risk Factors and Diabetes-Associated Complications

Blood pressure (SBP *z*-score, DBP *z*-score), lipids, (HDL-C, LDL-C, and triglycerides), and HbA1c were used as cardiovascular risk factor outcomes for this analysis [23] and assessed at SEARCH follow-up visit.

Diabetes-associated complications were defined by assessment at SEARCH visit.

1. Diabetic retinopathy was assessed via 45° color digital fundus images acquired with a nonmydriatic camera (Visucam Pro N, Carl Zeiss Meditech, Dublin, Ca). The Wisconsin Ocular Epidemiology Reading Center graded all images [24]. Retinopathy severity was based on the worse eye using established grading parameters and categorized as none, minimal nonproliferative, mild to moderate non-proliferative, and proliferative [25]. Diabetic retinopathy (yes/no) was then defined as having either mild, moderate, or proliferative retinopathy in at least one eye based on these grading parameters.
2. Peripheral neuropathy (yes/no) was defined as a score of >2 out of a total score of 8 on the Michigan Neuropathy Screening Instrument [26].
3. Cardiovascular autonomic dysfunction was assessed by heart rate variability utilizing the SphygmoCor-Vx device (AtCor Medical). Indices of heart rate variability derived from the electrocardiographic R-R intervals included: standard deviation (SD) of the intervals, root mean square differences of successive intervals, normalized high-frequency power, normalized low-frequency power, and the low-to-high frequency ratio [24]. Cardiovascular autonomic dysfunction (yes/no) was defined as participants having at minimum 3 of 5 heart rate variability abnormalities based on ≤5^th^ or ≥95^th^ percentile observed in age-and sex-matched control participants of the SEARCH Cardiovascular Disease Study [27].
4. Pulse wave velocity in the carotid to femoral segment (PWVf, continuous variable) was measured with the SphygmoCor device (AtCor Medical). The average of three recordings of PWVf was used, with higher PWVf indicating greater arterial stiffness [27].
5. Diabetic kidney disease (yes/no) was defined as the presence of albuminuria (≥30 *μ*g/mg of creatinine) or an estimated glomerular filtration rate ≤60 mL/min/1.73 m^2^ using the CKD-Epi equations with serum creatinine and cystatin C [28]. Samples were run on first-morning urine void samples, if not available, a spot sample was used [24].

### 2.6. Statistical Analysis

Data are presented as mean (SD) for continuous variables or frequency (%) for categorical variables. Basic demographic and clinical characteristics were compared between reported CD, seropositive for CD, and potentially undiagnosed CD to the comparison group (Tables 1). For frequencies, we used chi-square test for difference in proportions; Fishers exact test was used when expected counts were <5. For continuous measures we used a two-sample *t*-test.

To examine the relationship between CD status or tTGA levels with cardiovascular risk factors and diabetes-associated complications (Tables 2 and 3), complete case analysis was utilized on study outcomes and covariates to allow comparison across models and across unadjusted and adjusted estimates; except in the case of diabetic kidney disease, in which separate analysis was performed on the subset of the study population that had available urine samples. Linear regression models were utilized to estimate *ß*-coefficients for continuous outcomes (SBP, DBP, HDL-C, LDL-C, triglycerides, HbA1c, and PWVf) and a Poisson model with robust error variance was utilized to estimate relative risk (RR) for dichotomous outcomes (retinopathy, peripheral neuropathy, cardiovascular autonomic dysfunction, and diabetic kidney disease). For lipid outcomes (LDL-C, HDL-C, and triglycerides) adjusted models controlled for race and ethnicity and age at follow-up. For HbA1c and diabetes-associated complication outcomes, models controlled for race and ethnicity, age at follow-up, and age at diabetes diagnosis were adjusted. All models were adjusted for clinic site. These covariates were selected based on directed acyclic graph modeling [29] that were developed a priori based on the literature.

## 3. Results

The clinical characteristics of the 2,444 SEARCH individuals with type 1 diabetes included in this study stratified by CD status are presented in Table 1. The frequency of reported CD was 7%. Compared with participants with type 1 diabetes only (comparison group), participants with reported CD were younger at age of diabetes diagnosis (8.3 vs. 9.8 years, *p* < 0.01), more likely to be female (61.3% vs. 48.8%, *p* < 0.01) and differed regarding race and ethnicity (*p* < 0.01), with a higher percentage of those with reported CD consisting of NHW individuals. In addition, participants with reported CD had a lower BMI *z*-score (0.44 vs. 0.62, *p*=0.02) and SBP *z*-score (−0.84 vs. −0.66, *p*=0.02).

The frequency seropositive for CD (participants who did not report CD and had seropositive tTGA) was 4%. Compared with participants with type 1 diabetes only, participants seropositive for CD were younger at age of diabetes diagnosis (8.2 vs. 9.8 years, *p* < 0.01), more likely to be female (52.5% vs. 48.8%, *p*=0.02) and had lower HDL-C (52.4 vs. 56.1 mg/dL, *p* < 0.01).

Of these youth seropositive for tTGA with no reported CD, subanalysis was performed on those with tTGA ≥0.50 (≥10x ULN), suggesting potentially undiagnosed CD (*n* = 27). The frequency of tTGA seropositivity ≥10x ULN among those who did not report CD (*n* = 2444–168 = 2,276) was 1.2% (27/2,276). Of these participants with potentially undiagnosed CD (*n* = 27), twenty-three (*n* = 23) had complete data to perform complete case analysis. Participants with potentially undiagnosed CD (*n* = 23) had lower HDL-C (48.2 vs. 55.7 mg/dL, *p*=0.01) compared with participants with type 1 diabetes only.

We also examined coexisting autoimmune conditions amongst participants with type 1 diabetes by CD status (Supplementary Table (available here)). The frequency of hyperthyroidism, hypothyroidism, and Addison's disease were greater than two-fold higher in individuals with reported CD compared to the comparison group, youth with type 1 diabetes only (all *p* < 0.01). There were no differences in the frequency of autoimmune conditions between seropositive for CD and potentially undiagnosed CD compared to those with type 1 diabetes only.

We examined the relationship of CD status with cardiovascular risk factors and diabetes-associated complications (Table 2). In unadjusted analyses, the presence of reported CD was associated with lower SBP *z*-score (unadjusted *β*: −0.23 (95% Confidence Interval (CI): −0.41, −0.05)) and lower PWVf (unadjusted *β* −0.19 (95% CI: −0.40, −0.03)). After adjustment for covariates, neither association retained statistical significance. Reported CD was not significantly associated with any diabetes-associated complications. However, potentially undiagnosed CD was associated with higher risk of diabetic retinopathy in both unadjusted and adjusted models (adjusted RR 2.69 (95% CI: 1.66, 4.36)) compared with youth with type 1 diabetes only.

Lastly, we sought to determine if the tTGA levels correlated with cardiovascular risk factors. tTGA <0.05 in individuals with reported CD was associated with significantly lower HbA1c in both unadjusted and adjusted models (adjusted *β*: −0.55 (95% CI: −1.09, −0.02)) (Table 3). tTGA levels were not significantly associated with the remaining cardiovascular risk factors.

## 4. Discussion

This study describes the frequency and characteristics of reported CD and potentially undiagnosed CD and their relationship to cardiovascular risk factors and diabetes-associated complications in a large, geographically, and racial and ethnically diverse US population of youth with type 1 diabetes. In addition, this study assessed the relationship of tTGA levels with cardiovascular risk factors in youth with type 1 diabetes. Results of this study found that undiagnosed CD is likely present in youth with type 1 diabetes and those with potentially undiagnosed CD had a higher risk of diabetic retinopathy. Furthermore, individuals with CD and tTGA levels <0.05, suggesting controlled CD, are associated with lower HbA1c.

Youth with type 1 diabetes are at an increased risk for CD compared with the general population [3] in part due to shared HLA genetic risk [2]. The reported prevalence of CD in type 1 diabetes varies widely based on different data sources (1–16.4%) [6], likely secondary to studies performed in homogenous populations [30] and varying CD diagnostic criteria utilized [4, 6]. The largest prevalence study to date of CD in youth with type 1 diabetes was a cross-sectional study combining data from four registries (Registries from the US, Germany/Austria, the UK and Australia) and found a prevalence of 3.5% [4]. A systematic review of the prevalence of CD in children and adolescents with type 1 diabetes found a pooled prevalence of 5.1% [6]. In comparison, our findings demonstrated a 7% frequency of reported CD in youth with type 1 diabetes [4, 6]. Our data showing a greater risk of developing CD in youth diagnosed with diabetes at a younger age and that risk differs by race and ethnicity are consistent with prior work [4, 31]. Individuals with reported CD were more likely to be female than those with type 1 diabetes only, similar to the higher female prevalence of CD in the general population [30]. Reports on sex distribution of CD in youth with type 1 diabetes have been mixed [4, 32, 33], which may reflect the geographic, race, and ethnic makeup of the populations studied. The results reported here offer insight into the characteristics of CD in a multiethnic and race and geographically diverse adolescent type 1 diabetes population in the US.

Prevalence and epidemiologic studies likely underestimate CD in youth with type 1 diabetes since a majority of youth with CD is asymptomatic at the time of diagnosis [6] and diagnosis depends on local screening practices. In addition, some patients and families may choose not to undergo endoscopy, the gold standard for diagnosis, contributing to undiagnosed, and therefore unreported, CD in biopsy-confirmed prevalence studies. Our study design allowed for the unique opportunity to evaluate for potentially undiagnosed CD based on tTGA levels. Our data show 4% of our study population with type 1 diabetes had tTGA levels ≥0.05 indicating they were seropositive for CD without a reported CD diagnosis. While tTGA seropositivity does not indicate a true diagnosis of CD, as tTGA levels can be transiently elevated in individuals with type 1 diabetes and spontaneously normalize [34], it does raise the possibility that some youth with type 1 diabetes have undiagnosed CD. To this end, we found that amongst those with no reported diagnosis of CD, 1.2% had tTGA levels ≥10x ULN. To date, two published studies show individuals with tTGA levels ≥10x ULN all have diagnosed CD after intestinal biopsy in their study population [20, 21]. Furthermore, an additional study demonstrated a tTGA level ≥10x ULN (using the same assay as this study) at the time of biopsy had a 96% PPV for positive intestinal biopsy specimens [22]. In addition, all our study participants had diabetes duration >5 years at the time of tTGA assessment, ensuring tTGA elevation was not occurring at the time of diabetes diagnosis. Cases of potentially undiagnosed CD could be secondary to how study sites operationalize current CD screening recommendations for youth with type 1 diabetes [5]. Further studies are needed to explore potential barriers to CD screening and diagnosis in this population.

While the presence of diabetes-associated complications in individuals with type 1 diabetes and CD have been demonstrated in the adult and young adult populations [11, 35, 36], to our knowledge, no study to date has examined the risk of diabetes-associated complications in youth with type 1 diabetes by CD and potentially undiagnosed CD status. While reported CD was not found to significantly increase risk of diabetes-associated complications at this young age, individuals with potentially undiagnosed CD were found to have greater than 2.5× higher risk of diabetic retinopathy compared to those with type 1 diabetes only (comparison group with no reported CD and normal tTGA levels). Higher risk of diabetic retinopathy has been reported in multiple studies in adults with type 1 diabetes and CD [11, 35, 36]. Leeds et al. found that adults with type 1 diabetes and newly diagnosed CD had a higher prevalence of advanced retinopathy and nephropathy [35]. Rohrer et al. found the adjusted risk for both retinopathy and nephropathy were higher in patients with type 1 diabetes and CD versus those without CD, and both conditions occurred earlier [11]. The increased risk for the microvascular complication of diabetic retinopathy in individuals with type 1 diabetes with both diagnosed, and now potentially undiagnosed, CD suggests that the concurrent disease process of CD may negatively impact the microvasculature in individuals with type 1 diabetes. The mechanisms behind type 1 diabetes and CD on the microvasculature is unclear, though inflammation [8, 37, 38] has been suggested to play a role. In addition, the higher risk specifically of the microvascular complication of retinopathy, as demonstrated here in youth with potentially undiagnosed CD and in adults with CD and type 1 diabetes [11, 35], remains undetermined. A potential contributing factor specifically for retinopathy, however, may be reduced vitamin A levels secondary to malabsorption in the setting of CD [39]. Vitamin A plays an important role in the retina [40] and malnutrition retinopathy has been described in CD [39], although this warrants further investigation. Lastly, studies specifically assessing outcomes in those with undiagnosed CD are limited, but in the general adult population, undiagnosed CD was associated with a nearly 4-fold increased risk of death over seronegative individuals [41]. Therefore, prior works and our study results add credence to the role of CD and potentially undiagnosed CD, as a potential added risk factor for increased morbidity [42], especially in the already higher risk type 1 diabetes population. Concerningly, this risk may begin at a young age. Future studies are needed to determine the mechanisms in which the combined disease process increases risk for these outcomes.

Finally, we sought to assess the relationship between tTGA levels with cardiovascular risk factors. CD [10] and type 1 diabetes [43] are both associated with increased cardiovascular risk, and combined disease appears to accelerate the atherosclerotic process [9]. In addition, the role tTGA levels and CD control plays in cardiovascular risk is not fully understood [35, 44]. In analysis focused on tTGA levels, we demonstrated that tTGA levels' suggesting controlled CD was associated with lower HbA1c. Similar findings were demonstrated in another large study of youth with type 1 diabetes [44], which found that individuals with CD who had normalization of CD antibodies had lower HbA1c than individuals with CD who had positive CD antibody status and those with type 1 diabetes only. It is possible that any difference detected in HbA1c in those with controlled CD reflects confounding by an individual's overall health behavior and/or medical adherence.

Strengths of this study include analysis of a diverse race and ethnic cohort, standardized tTGA measurements, and the ascertainment of tTGA levels on 2,444 youth with type 1 diabetes to assess the impact of tTGA levels on risk factors and identify potentially undiagnosed CD. An additional strength was the ability to simultaneously assess CD status, tTGA levels, and an array of cardiovascular risk factors and diabetes-associated complications in a large cohort of youth with type 1 diabetes. Limitations of this study are mainly centered on the data collected in SEARCH pertaining to CD. The original design of the SEARCH study did not include comprehensive assessment of CD or dietary data, including adherence to a gluten-free diet. As a result, CD status was based on report via questionnaire without the ability to verify if they have biopsy proven CD. However, to internally validate reported CD in our subset of the study population, we were able to review individuals who reported CD at a single site (Ohio) only for biopsy proven CD. We found that of the 21 who reported CD, 18 had biopsy proven CD, one is being worked up for CD, and one has not undergone a biopsy but avoids gluten altogether now, demonstrating reliability of self-report data. Lastly, IgA levels were not obtained on all individuals as the prevalence of IgA deficiency in the US ranges from 1 : 233–1 : 1,000 [45], indicating IgA deficiency in potentially 2–10 participants in this study. This frequency of IgA deficiency does not influence the statistical outcomes presented here and, if anything, would cause us to miss a CD case, underestimating our positive findings.

In conclusion, we demonstrate that there is a potential for undiagnosed CD in youth with type 1 diabetes. Furthermore, potentially undiagnosed CD was associated with a higher risk of diabetic retinopathy. In addition, tTGA levels may be associated with the cardiovascular risk factor HbA1c. These findings indicate the importance of routine screening for CD in type 1 diabetes in youth.

## Figures and Tables

**Table 1:** Descriptive and clinical characteristics of youth with type 1 diabetes by celiac disease status.

Variables	Comparison group: type 1 diabetes only^a^	Reported CD^b^	*p* value for ^b^vs^a^	Seropositive for CD^c^	*p* value for ^c^vs^a^	Potentially undiagnosed CD^d^	*p* value for ^d^vs^a^
*N* (%)	2175 (89.0)	168 (6.9)		101 (4.1)		23 (1.0)	
Age at follow-up, mean (SD)	17.5 (4.5)	16.0 (4.3)	**<0.01**	15.7 (4.6)	**<0.01**	15.7 (4.0)	0.06
Age at diabetes diagnosis, mean (SD)	9.8 (4.3)	8.3 (4.0)	**<0.01**	8.2 (4.4)	**<0.01**	8.3 (3.9)	0.10
Diabetes duration in months, mean (SD)	101.9 (24.5)	101.6 (23.1)	0.89	99.0 (24.5)	0.25	96.3 (23.5)	0.28
Female sex, *n* (%)	1061 (48.8)	103 (61.3)	**<0.01**	53 (52.5)	**0.02**	15 (65.2)	0.12
*Race/ethnicity*, *n* (%)							
Black	251 (11.5)	4 (2.4)		5 (5.0)		0 (0)	0.14
Hispanic	310 (14.3)	23 (13.7)	**<0.01**	11 (10.9)	0.12	2 (8.7)	
Non-Hispanic White	1486 (68.3)	137 (81.6)		79 (78.2)		21 (9.3)	
Other	128 (5.9)	4 (2.4)		6 (5.9)		0 (0)	
*Parental education*, *n* (%)							
<High school graduate	96 (4.5)	2 (1.2)		3 (3.0)		0 (0)	0.86
High school graduate	348 (16.2)	20 (12.2)	**0.04**	16 (16.2)	0.90	3 (13.0)	
Some college	690 (32.1)	49 (29.9)		31 (31.3)		7 (30.4)	
Bachelor's degree or more	1013 (47.2)	93 (56.7)		49 (49.5)		13 (56.5)	
*Insurance status*—*n* (%)							
Private	1497 (69.5)	124 (74.7)		71 (70.3)		16 (69.6)	0.19
Medicaid/medicare	470 (21.8)	29 (17.5)	0.28	17 (16.8)	0.27	3 (13.0)	
Other	108 (5.0)	10 (6.0)		9 (8.9)		3 (13.0)	
None	79 (3.7)	3 (1.8)		4 (4.0)		1 (4.4)	
BMI *z*-score, mean (SD)	0.62 (0.96)	0.44 (0.90)	**0.02**	0.52 (0.91)	0.29	0.24 (1.01)	0.06
SBP *z*-score, mean (SD)	−0.66 (0.98)	−0.84 (1.01)	**0.02**	−0.68 (1.01)	0.83	−0.68 (0.86)	0.92
DBP *z*-score, mean (SD)	0.24 (0.88)	0.14 (0.83)	0.18	0.12 (0.89)	0.18	0.19 (0.77)	0.80
HbA1c % at follow-up, mean (SD)	9.2 (1.9)	8.9 (1.9)	0.09	9.1 (1.8)	0.64	8.1 (1.9)	0.14
HbA1c mmol/mol at follow-up, mean (SD)	77 (7)	74 (7)	0.09	76 (7)	0.64	65 (7)	0.14
HDL-C mg/dL, mean (SD)	55.7 (14.1)	56.1 (12.1)	0.77	52.4 (11.4)	**<0.01**	48.2 (11.9)	**0.01**
LDL-C mg/dL, mean (SD)	97.4 (29.1)	94.6 (28.6)	0.23	93.5 (23.3)	0.19	97.9 (89.9)	0.94
Triglycerides mg/dL, mean (SD)	94.1 (77.7)	85.2 (52.8)	**0.05**	84.1 (60.3)	0.12	87.6 (55.1)	0.69

SD = standard deviation; CD = celiac disease. ^a^Comparison group: participants with type 1 diabetes and no evidence of CD (negative questionnaire response and seronegative tTGA <0.05). ^b^Reported CD: participants with type 1 diabetes and reported CD (positive questionnaire response, regardless of tTGA level). ^c^Seropositve for CD: participants with type 1 diabetes and no reported CD (negative questionnaire response) and seropositive tTGA (tTGA ≥0.05). ^d^Potentially undiagnosed CD: participants with type 1 diabetes and no reported CD (negative questionnaire response) and seropositive tTGA ≥10x ULN (tTGA ≥0.50) are a subset of ^c^seropositive for CD.

**Table 2:** Cardiovascular risk factors and diabetes-associated complications in type 1 diabetes by celiac disease status.

Outcomes	Comparison group	Reported CD^c^	Potentially undiagnosed CD^d^	Reported CD^c^	Potentially undiagnosed CD^d^
	Type 1 diabetes only^b^				
		*Unadjusted—β (95% CI)*		*Adjusted* ^*a*^ *—aβ (95% CI)*	
*n*	1531	121	23	121	23
SBP *z*-score	Referent	**−0.23 (-0.41, −0.05)**	0.04 (−0.43, 0.51)	−0.14 (−0.31, 0.03)	−0.05 (−0.49, 0.38)
DBP *z*-score	Referent	−0.04 (−0.20, 0.12)	−0.01 (−0.43, 0.40)	−0.03 (−0.20, 0.13)	0.04 (−0.37, 0.45)
HDL-C (mg/dL)	Referent	0.70 (−1.87, 3.27)	−6.00 (−12.67, 0.67)	0.54 (−2.02, 3.11)	−6.47 (−13.07, 0.14)
LDL-C (mg/dL)	Referent	−2.09 (−7.51, 3.34)	3.29 (−10.63, 17.20)	−1.14 (−6.61, 4.33)	4.42 (−9.49, 18.34)
Triglycerides (log) (mg/dL)	Referent	−0.05 (−0.15, 0.05)	−0.05 (−0.31, 0.21)	−0.03 (−0.13, 0.07)	−0.01 (−0.27, 0.24)
HbA1c (%)	Referent	−0.29 (−0.65, 0.06)	−0.76 (−1.66, 0.15)	−0.17 (−0.51, 0.17)	−0.67 (−1.54, 0.21)
PWVf (m/s)	Referent	**−0.19 (-0.40, −0.03)**	−0.51 (−1.07, 0.05)	0.01 (−0.19, 0.20)	−0.24 (−0.75, 0.26)

		*Unadjusted—RR (95% CI)*		*Adjusted* ^*a*^ *—aRR (95% CI)*	
Retinopathy	Referent	0.79 (0.53, 1.18)	**1.85 (1.04, 3.30)**	1.00 (0.67, 1.52)	**2.69 (1.66, 4.36)**
Peripheral neuropathy	Referent	1.51 (0.85, 2.66)	Not estimable	1.69 (0.98, 2.92)	Not estimable
Cardiovascular autonomic dysfunction	Referent	0.97 (0.57, 1.65)	0.52 (0.08, 3.50)	1.06 (0.61, 1.82)	0.62 (0.10, 4.05)
Diabetic kidney disease^e^	Referent	1.11 (0.52, 2.33)	1.02 (0.15, 6.86)	1.30 (0.61, 2.75)	1.27 (0.19, 8.73)

CD = celiac disease; PWVf = pulse wave velocity carotid-femoral; CI = confidence interval. ^a^SBP and DBP were adjusted for clinic site; lipid measurements (LDL-C, triglycerides and HDL-C) adjusted for race and ethnicity, age at follow-up and clinic sites; all other cardiovascular risk factors and diabetes-associated complications adjusted for age at diabetes diagnosis, age at follow-up, race and ethnicity, and clinic sites. ^b^Type 1 diabetes only: participants with type 1 diabetes and no evidence of CD (negative questionnaire response and seronegative tTGA <0.05). ^c^Reported CD: participants with type 1 diabetes and reported CD (positive questionnaire response, regardless of tTGA level). ^d^Potentially undiagnosed CD: participants with type 1 diabetes and no reported CD (negative questionnaire response) and seropositive tTGA ≥10x ULN (tTGA ≥0.50). ^e^Analysis for diabetic kidney disease was performed on a subset of the SEARCH population who had urine samples available *n* = 1553.

**Table 3:** tTGA levels and cardiovascular risk factors in type 1 diabetes.

Outcomes	Comparison group	Unadjusted—*β* (95% CI)			Adjusted^a^—a*β* (95% CI)		
	Type 1 diabetes only^b^	Any participant with high tTGA (≥0.50) elevation^c^	Any participant with intermediate (≥0.05 and <0.50) tTGA elevation^d^	Participants with reported CD and controlled tTGA <0.05^e^	Any participant with high tTGA (≥0.50) elevation^c^	Any participant with intermediate (≥0.05 and <0.50) tTGA elevation^d^	Participants with reported CD and controlled tTGA <0.05^e^
*n*	1531	47	96	46	47	96	46
SBP *z*-score	Referent	−0.10 (−0.39, 0.18)	−0.22 (−0.43. −0.02)	−0.13 (−0.41, 0.16)	−0.09 (−0.37, 0.17)	−0.12 (−0.31, 0.07)	−0.05 (−0.31, 0.22)
DBP *z*-score	Referent	0.02 (−0.24, 0.28)	−0.05 (−0.23, 0.13)	−0.07 (−0.32, 0.18)	0.01 (−0.24, 0.27)	−0.04 (−0.22, 0.15)	−0.05 (−0.30, 0.20)
LDL-C (mg/dL)^f^	Referent	2.75 (−5.79, 11.28)	−3.07 (−9.10, 2.97)	−5.74 (−14.21, 2.72)	3.39 (−5.15, 11.93)	−2.16 (−8.23, 3.91)	−4.80 (−13.29, 3.69)
TG (log) (mg/dL)	Referent	0.04 (−0.11, 0.20)	−0.07 (−0.19, 0.04)	−0.13 (−0.28, 0.03)	0.06 (−0.09, 0.22)	−0.06 (−0.17, 0.05)	−0.11 (−0.27, 0.05)
HDL-C (mg/dL)	Referent	−3.47 (−7.58, 0.63)	−0.82 (−3.69, 2.04)	2.45 (−1.58, 6.47)	−3.77 (−7.82, 0.29)	−0.99 (−3.85, 1.87)	1.99 (−2.01, 5.99)
HbA1c (%)	Referent	0.17 (−0.39, 0.73)	−0.02 (−0.41, 0.37)	**−0.61 (-1.16, −0.06)**	0.25 (−0.30, 0.79)	0.10 (−0.28, 0.49)	**−0.55 (-1.09, −0.02)**

CD = celiac disease; SBP = systolic blood pressure; DBP = diastolic blood pressure; TG = triglycerides; CI = confidence interval. ^a^SBP and DBP were adjusted for clinic site; lipid measurements (LDL-C, triglycerides and HDL-C) adjusted for race and ethnicity, age at follow-up and clinic site; all other cardiovascular risk factors and diabetes-associated complications adjusted for age at diabetes diagnosis, age at follow-up, race and ethnicity, and clinic site. ^b^Participants with type 1 diabetes and no evidence of CD (negative questionnaire response and seronegative tTGA <0.05). ^c^Participants with type 1 diabetes and elevated tTGA 10x ULN (≥0.50) regardless of CD questionnaire response, “high tTGA elevation.” ^d^Participants with type 1 diabetes and elevated tTGA (≥0.05 and <0.50) regardless of CD questionnaire response, “intermediate tTGA elevation.” ^e^Participants with type 1 diabetes and reported CD (positive questionnaire response) and normal tTGA (<0.05), “Controlled CD.” ^f^Missing *n* = 58.

## Data Availability

The datasets supporting the current study are available at the NIH and are available from the corresponding author on request.

## Supplementary Materials

Supplementary Materials Supplementary table: co-autoimmunity describes the frequency of coexisting autoimmune conditions amongst participants with type 1 diabetes by the CD status.
