# Supplementary material for: The Frequency of Undiagnosed Celiac Disease in Youth with Type 1 Diabetes and Its Association with Diabetic Retinopathy: The SEARCH for Diabetes in Youth Study
Source: Pediatr Diabetes. 2023 May 29;2023:9038795. doi: 10.1155/2023/9038795 (PMC11753297; doi:10.1155/2023/9038795)
Supplement: Supplementary Materials — Supplementary table: co-autoimmunity describes the frequency of coexisting autoimmune conditions amongst participants with type 1 diabetes by the CD status. [file 9038795.f1.pdf]

Supplementary Table. Co-existing Autoimmunity in Youth with Type 1 Diabetes by Celiac Disease Status

Factor	Comparison group: 1 Diabetes Only[a]	Reported CD[b]	p-value for [b] vs [a]	Seropositive for CD[c]	p-value for [c] vs [a]	Potentially Undiagnosed CD[d]	p-vale for [d] vs [a]
N (%)	2175 (89.0)	168 (6.9)		101 (4.1)		23 (1.0)	
Hyperthyroidism – n (%)	51 (2.3)	14 (8.3)	<0.01	4 (4.0)	0.31	1 (4.4)	0.43
Hypothyroidism – n (%)	139 (6.4)	24 (14.3)	<0.01	8 (7.9)	0.54	1 (4.4)	1.0
Addison's Disease – n (%)	7 (0.3)	5 (3.0)	<0.01	0 (0.0)	1.00	0 (0)	1.0
Vitiligo - n (%)	43 (2.0)	7 (4.2)	0.09	3 (3.0)	0.46	2 (8.7)	0.08

CD=Celiac Disease

[a]Comparison group: Participants with type 1 diabetes and no evidence of CD (negative questionnaire response and serologic negative)

[b]Reported CD: Participants with type 1 diabetes and reported CD (positive questionnaire response, regardless of tTGA level)

[c]Seropositive for CD: Participants with type 1 diabetes and no reported CD (negative questionnaire response) and seropositive tTGA (tTGA ≥0.05)

[d]Potentially Undiagnosed CD: Participants with type 1 diabetes and no reported CD (negative questionnaire response) and seropositive tTGA ≥10x ULN (tTGA ≥0.50) are a subset of [c]Seropositive for CD
